# Does COVID-19 vaccine exacerbate rotator cuff symptoms? A prospective study

**DOI:** 10.1186/s12891-023-06660-y

**Published:** 2023-07-04

**Authors:** Servet İğrek, İbrahim Ulusoy, Aytek Hüseyin Çeliksöz

**Affiliations:** 1 Department of Orthopaedics and Traumatology, Selahaddin Eyyubi State Hospital, Diyarbakır, Turkey; 2 Department of Orthopaedics and Traumatology, Eskisehir City Hospital, Eskişehir, Turkey

**Keywords:** SIRVA, COVID-19, Vaccine, Subacromial-subdeltoid bursitis, Pain, Shoulder

## Abstract

**Background:**

Shoulder injury related to vaccine administration (SIRVA) is a rare but increasing complication after vaccination. The aim of this study was to increase awareness of post-vaccination shoulder pain and to investigate the effect of the clinical condition of the shoulder before vaccination on the loss of function that may occur after vaccination.

**Methods:**

This prospective study included 65 patients aged > 18 years who were diagnosed with unilateral shoulder impingement and/or bursitis. The first vaccination was performed on the shoulders with rotator cuff symptoms, then the second vaccination was performed on healthy shoulders of same patients as soon as the health system allowed. Pre-vaccination MRI of the symptomatic shoulders of the patients was performed and VAS, ASES and Constant scores were evaluated. At 2 weeks after vaccination of the symptomatic shoulder, scores were reassessed. For the patients with changes in the scores, MRI was performed again and the treatment of all patients was started. A second vaccination was given to asymptomatic shoulders and the patients were recalled two weeks later and their scores were evaluated.

**Results:**

After vaccination, the symptomatic shoulder of 14 patients was affected. No clinical changes were observed in the asymptomatic shoulders after vaccination. The VAS scores of the symptomatic shoulders evaluated after vaccination were significantly higher than the scores evaluated before vaccination (*p* = 0.001). The ASES and Constant scores of symptomatic shoulders evaluated after vaccination were significantly decreased compared to the scores evaluated before vaccination (*p* = 0.001).

**Conclusions:**

Exacerbation of symptoms may occur if symptomatic shoulders are vaccinated. Before vaccination, a detailed anamnesis should be taken from the patients and vaccination should be performed to the asymptomatic side.

## Introduction

As a result of the COVID-19 pandemic, the number of people vaccinated worldwide has increased compared to previous years. With the increase in vaccinations, there has also been an increase in the number of patients admitted to the hospital with shoulder pain in our daily clinical practice. Administration of vaccines to the shoulder area has been associated with a specific side effect known as "shoulder injury related to vaccine administration" (SIRVA), which is particularly associated with influenza vaccines [1, 2]. SIRVA may include shoulder pain and limited range of motion with many findings including adhesive capsulitis, tendinitis, subacromial-subdeltoid bursitis, and partial rotator cuff tears [3]. The literature related to SIRVA and COVID-19 vaccines is very limited, consisting only of case reports [4–7].

According to our observations, some patients who were being followed up with conservative treatment for reasons such as subacromial-subdeltoid bursitis, or partial rotator cuff tear, were determined with exacerbation in the symptomatic shoulder after vaccination. It has been determined that there is not enough data in the orthopedic literature on this subject and there is not enough awareness [8]. The aim of this study was to clinically and radiologically evaluate the post-COVID-19 vaccination symptoms of such patients, who are frequently seen in orthopedic practice, to investigate the differences between asymptomatic and symptomatic shoulders after vaccination, and to raise awareness of shoulder pain increasing after vaccination.

## Materials and method

This prospective, single-centre study was conducted on patients who presented at the Orthopedics Clinic with shoulder pain between January and September 2021. Approval for the study was granted by the Institutional Review Board. Informed consent was provided by all patients. The study was conducted in accordance with the principles of the Helsinki Declaration.

The study inclusion criteria were as follows:

1. Patients aged > 18 years
2. Having not been vaccinated before and agreeing to be vaccinated
3. A diagnosis of unilateral impingement syndrome or subacromial-subdeltoid bursitis
4. No previously shoulder pathology at the contralateral side
5. Acceptance of the Pfizer-BioNTech COVID-19 (BNT162b2) vaccine

**Fig. 1:**
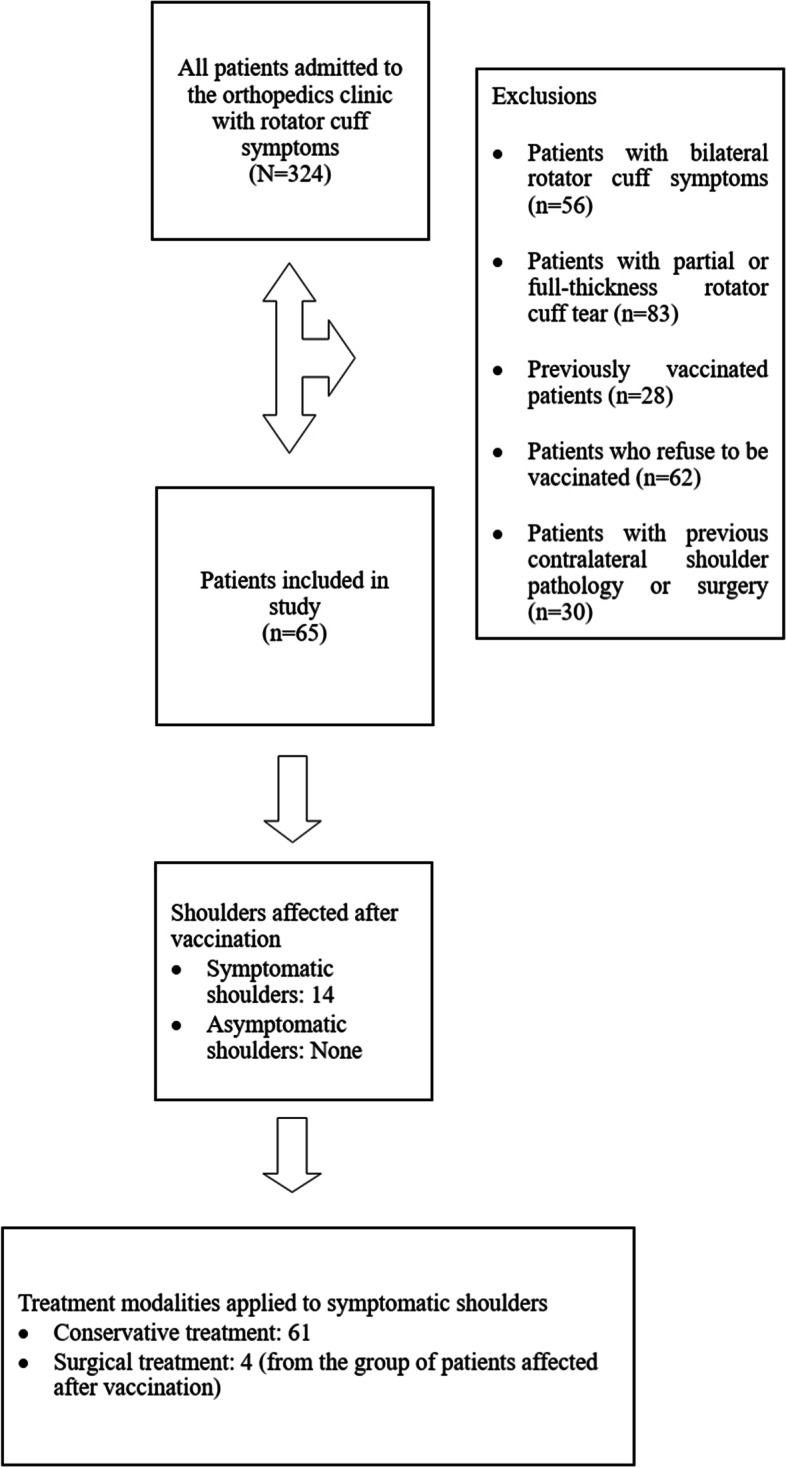
Flow chart

The study exclusion criteria were defined as age < 18 years, patients who refused to be vaccinated, had been vaccinated before, or did not accept the Pfizer-BioNTech COVID-19 (BNT162b2) vaccine, patients with bilateral shoulder problems, with partial or full-thickness rotator cuff tears, acromioclavicular joint pain, glenohumeral joint pain, pain due to instability or post-traumatic pain, and patients with a history of shoulder surgery. See flow chart in Fig. 1.

After anamnesis and physical examination of all patients who presented at the clinic with shoulder pain, an magnetic resonance imaging (MRI) was performed on the symptomatic shoulder. Evaluations were then made with the visual analog scale (VAS), American Shoulder and Elbow Surgeons (ASES) score and the Constant score of both the symptomatic and asymptomatic shoulders of all the patients who agreed to participate in the study and met the conditions. The Pfizer-BioNTech COVID 19 (BNT162b2) vaccine was applied to the symptomatic shoulders. The patients were re-evaluated at 2 weeks after the vaccination, and the VAS, ASES and Constant scores of the vaccinated symptomatic shoulders of all patients were re-evaluated. MRI was performed again in all patients who had an increase in shoulder pain and a change in their scores. MR images were evaluated by two authors experienced in shoulder.After the evaluations, conservative treatment was started for all patients and patients with persistent symptoms were treated with surgery. Second vaccinations were applied to the healthy and asymptomatic shoulders of all patients when deemed appropriate by the national health system. All vaccinations were administered by the same healthcare staff as described in the literature [9, 10]. At 2 weeks after the second vaccination, all patients were recalled and the VAS, ASES and Constant scores of healthy shoulders were re-evaluated.

### Statistical analysis

Data obtained in the study were analyzed statistically using NCSS (Number Cruncher Statistical System) 2007 software (Kaysville, Utah, USA). Descriptive statistical methods (mean, standard deviation, median, frequency, percentage, minimum, maximum) were used in the data evaluations. The Dependent groups t-test was used for comparisons within the group of normally distributed quantitative variables and the Wilcoxon signed-ranks test was used for quantitative variables that did not show normal distribution. A value of *p* < 0.05 was accepted as statistically significant.

## Results

Evaluation was made of a total of 65 patients who met the criteria, comprising 20 males and 45 females with an average age of 52.99 ± 10.18 years (range, 34 to 72 years).

It was determined that 14 (21.5%) patients were affected after vaccination. There was no statistically significant difference between the distribution of affected and unaffected patients after vaccination according to age and gender (*p* > 0.05).

The VAS scores of symptomatic shoulders evaluated after vaccination were significantly higher than the pre-vaccination VAS scores (*p* = 0.001). No difference was found between the pre and post-vaccine VAS scores of asymptomatic shoulders (Table 1).

**Table 1:** VAS scores before and after vaccination

	**Shoulder**	
	**Symtomatic**	**Asymptomatic**
**VAS before vac**	5,85 ± 1,21	0 ± 0
**VAS after vac**	6,20 ± 1,39	0 ± 0
***p value***	***0,001***	***-***

Paired Sample T Test *p* < 0,01

VAS Visual analog scale, *Vac* Vaccine

The ASES and Constant scores of symptomatic shoulders evaluated after vaccination were significantly decreased compared to the ASES and Constant scores evaluated before vaccination (*p* = 0.001). There was no difference between the pre and post-vaccination ASES and Constant scores of asymptomatic shoulders (Table 2).

**Table 2:** ASES and Constant scores before and after vaccination

	**Shoulder**	
	**Symtomatic**	**Asymptomatic**
**ASES before vac**	48,49 ± 7,85	100 ± 0
**ASES after vac**	46,81 ± 8,43	100 ± 0
**Constant before vac**	56,61 ± 7,75	100 ± 0
**Constant after vac**	54,90 ± 8,38	100 ± 0
***p value***	***0,001***	**-**

Wilcoxon Signed Rank Test *p* < 0,01

*ASES* American shoulder and elbow surgeons score, *Vac* Vaccine

Corticosteroid injection was applied to the patients with symptoms persisting after non-steroidal anti-inflammatory therapy and 3 months of physical therapy. Surgical intervention was performed in 4 (6.1%) patients whose symptoms persisted after about 4 months of conservative treatment. All patients who underwent surgical treatment were in the group of patients affected after vaccination. All patients continued physical therapy for 3 months after surgery.

**Fig. 2:**
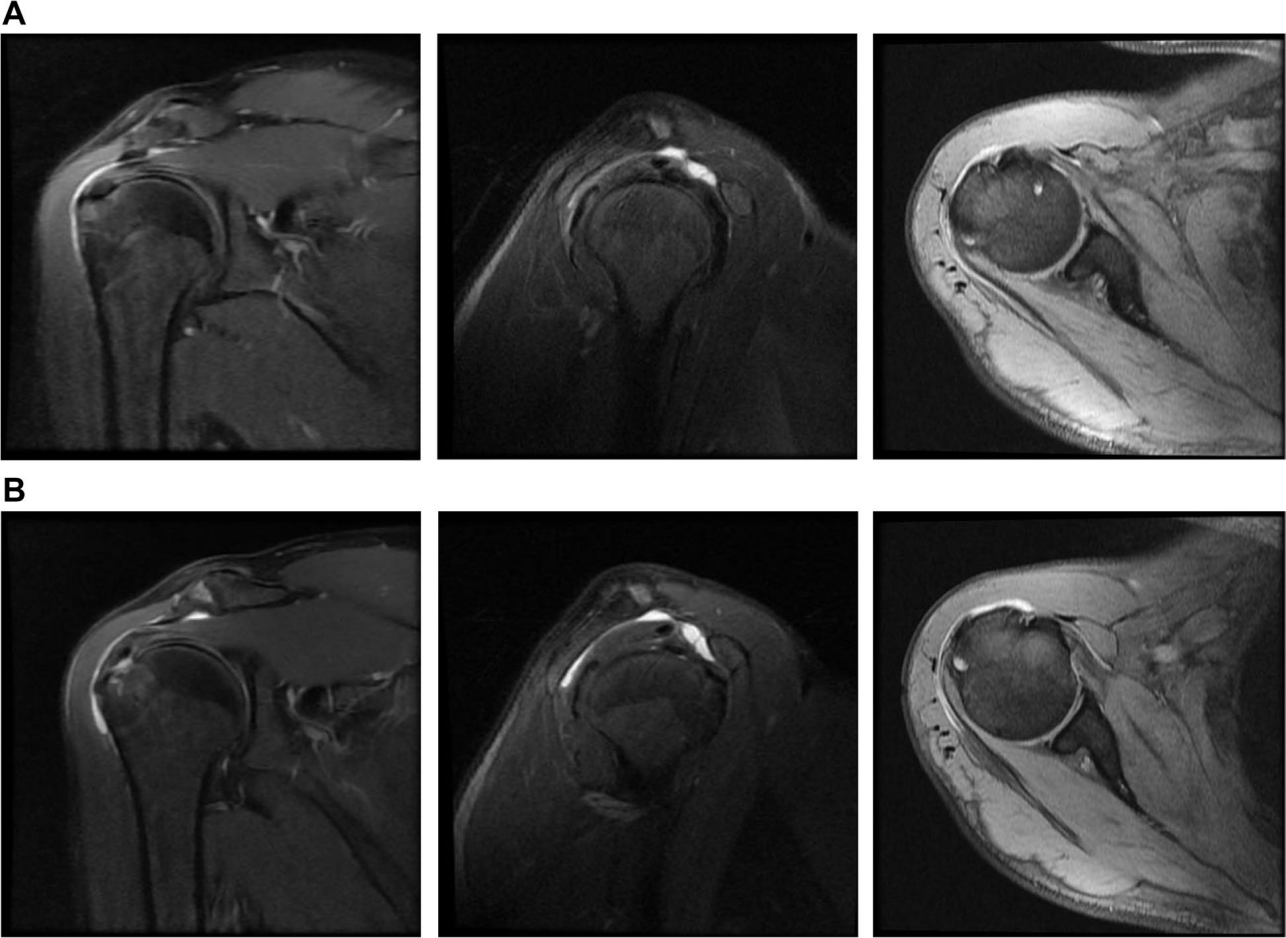
MRI images of a 47-year-old male patient. **A** Images of bursitis with intact rotator cuff before vaccination. **B** Post-vaccination images show increased bursitis and partial rotator cuff tear

The appearance of subacromial-subdeltoid bursitis were determined to have increased radiologically in the follow-up MRIs of the affected patients after the vaccination. Post-vaccine partial rotator cuff tear was detected in one patient (Fig. 2).

## Discussion

SIRVA is a diagnosis that occurs 48 h after vaccination in patients without previous shoulder pain, inflammation or loss of function, and includes a series of findings together with long-lasting pain and dysfunction [11]. Although the pathogenesis of SIRVA is not fully understood, the most accepted theory is that it occurs as a result of a prolonged inflammatory response after the vaccine is injected into the submuscular tissues by passing through the deltoid muscle [3, 11, 12]. Bodor et al. [9] reported that bursitis, tendinitis and capsulitis may develop after vaccination to the subdeltoid region due to the connection of the subdeltoid bursa with the subacromial bursa. Reported cases of SIRVA mostly include rotator cuff tendinopathy and/or bursitis without a tear, but may occasionally include complete or partial rotator cuff tear [7, 12]. All of the literature on this subject consists of retrospective studies and case reports [13]. In those cases, none of the patients diagnosed with SIRVA in the past were evaluated clinically or radiologically before vaccination and the absence of shoulder complaints before vaccination was based on patient statements. In a case report of a patient who was found to have SIRVA after COVID-19 vaccination, it was stated that the patient had no shoulder pain before vaccination and had pain in the right shoulder 72 h after vaccination. Glenohumeral joint arthrosis was detected on the shoulder X-ray of the patient [6]**.** Therefore, it can be considered that in this shoulder with arthrosis, the pain felt by the patient could possibly be secondary to strenuous activities before vaccination. The taking of a detailed anamnesis before vaccination will reveal such situations and the other shoulder can be vaccinated. In another case report, cortical irregularities and subcortical sclerosis were detected in the greater tubercle on the X-ray taken of the shoulder of a patient who developed SIRVA after vaccination, and bursitis and rotator cuff tears were detected in the ultrasound images. It is unknown whether all these radiological findings occurred after vaccination or if some were present before vaccination [7]**.** In a case report published by Rodrigues et al., it was reported that SIRVA developed in a patient after vaccination with an inappropriate technique, and subacromial-subdeltoid bursitis and rotator cuff tendinopathy were detected on MRI and ultrasound images of the patient. Although the patient was not questioned about shoulder complaints before vaccination, but it was stated that he had not received any treatment for inflammatory arthropathy in the past [4]. In this context, the current study, due to its prospective nature, provided the opportunity to evaluate all patients clinically and radiologically before vaccination and enabled the determination of changes that occurred after vaccination. The clinical and radiological exacerbations after vaccination in shoulders that were symptomatic before vaccination, and the absence of any clinical changes after vaccination in asymptomatic shoulders, unlike symptomatic shoulders, revealed the importance of the clinical status of the pre-vaccination shoulder.

The vaccination technique is very important. Vaccination applied higher than the recommended level, deep enough to pass through the muscle and reach the bursal tissues, or the direction of the needle during vaccination may be the cause of SIRVA and improper technique may reduce the effectiveness of the vaccine [9, 10]. Vaccination with an inappropriate technique has been shown to be the cause of SIRVA developing after COVID-19 vaccination in reported cases in literature [4–7]. In the current study, all the vaccines were administered by the same healthcare staff, who were trained on the subject, using the correct method, so that all problems that may arise from inappropriate technique were excluded. The fact that all shoulders that were asymptomatic before vaccination did not develop any symptoms after vaccination showed the importance of correct technique. Despite the use of an appropriate technique for shoulders that were symptomatic before vaccination, the increase in complaints after vaccination in some patients revealed the importance of vaccination with the correct technique, as well as the clinical condition of the shoulder during vaccination.

The initial treatment for patients diagnosed with SIRVA is physical therapy and NSAIDs, and subacromial, subdeltoid corticosteroid injections can be added to the treatment for patients with ongoing symptoms [11, 12, 14]. Some patients diagnosed with SIRVA for whom conservative treatment has failed may require surgical intervention. While Hesse et al. [3] stated that surgery was required in 32.6% of patients, Hibbs et al. [14] reported that surgery was performed in 2.9% of patients. There is no standard surgical treatment method for SIRVA, and various surgical methods such as subacromial decompression, joint debridement, rotator cuff repair, bursectomy, and manipulation under anesthesia can be applied according to MRI and examination findings [3]. In the current literature, 3 case reports have been published in which the symptoms regressed with arthroscopic debridement and physical therapy after the diagnosis of SIRVA and unsuccessful conservative treatment [9, 15, 16]. In the current study, conservative treatment was sufficient in patients whose symptoms did not change after vaccination. Surgical intervention was performed in 4 (28.5%) patients from the group with increased symptoms after vaccination. Subacromial debridement and bursectomy were applied to the patients who underwent surgery and cuff repair was performed in one patient with partial rotator cuff tear (Fig. 2). After surgery, all patients received physical therapy and their treatment was completed. Consistent with the literature, it was observed in this study that the need for surgical treatment increased after SIRVA.

Limitations of this study were primarily the small number of patients and the use of a single type of vaccine. Different symptoms may be seen in studies of larger numbers of patients and different vaccines. In this study, a single type of vaccine was used to ensure standardization. It should be noted as an another limitation of the study is that it was not conducted in a healthy population without previous shoulder problems. However, the most important aspect of the study is that it was a prospective study. Studies involving larger patient groups and different vaccine types may contribute to more valid results.

## Conclusion

SIRVA is a condition that will be seen more frequently in the future with the increase in vaccinations. The most important aspect of this subject is to prevent the development of SIRVA, which can be achieved by increase the awareness of healthcare professionals. Exacerbation of symptoms may occur if symptomatic shoulders are vaccinated. Before vaccination, a detailed anamnesis should be taken from the patients. Vaccination should be performed with the correct technique to the asymptomatic side. Thus, the possibility of post-vaccination shoulder dysfunction that may require surgery can be minimized.

## Data Availability

Data sharing is not applicable to this article as no datasets were generated or analysed during the current study. Servet İğrek should be contacted if someone wants to request the data from this study.
